# Supplementary material for: Unveiling health disparities: Diagnostic prevalences in a transgender cohort versus matched controls
Source: PLoS One. 2025 Aug 6;20(8):e0329849. doi: 10.1371/journal.pone.0329849 (PMC12327606; doi:10.1371/journal.pone.0329849)
Supplement: S1 Table — Prevalence of medical clinical phenotypes for TGD cases and population controls, and association of phenotypes with TGD group membership. (DOCX) [file pone.0329849.s001.docx]

**S1 Table. Prevalence of medical clinical phenotypes for TGD cases and population controls, and association of phenotypes with TGD group membership**

| **Clinical Phenotypes** | **TGD Cases**  **(N=6664)** | **Population Controls (N=64124)** | **Prevalence Ratio**  **(PR)**  **(95% CI)** | ***p*-value^a^** | **Adjusted PR^b^**  **(95% CI)** | ***p*-value^a^** |
| --- | --- | --- | --- | --- | --- | --- |
| **Endocrine/Metabolic** |  |  |  |  |  |  |
| Other endocrine disorders | 1100 (17%) | 932 (1%) | 11.36 (10.45-12.35) | <0.001 | 7.65 (7.01-8.35) | <0.001 |
| Anorexia | 219 (3%) | 775 (1%) | 2.72 (2.35-3.15) | <0.001 | 1.73 (1.49-2.00) | <0.001 |
| Other nutritional deficiency | 158 (2%) | 586 (1%) | 2.59 (2.18-3.09) | <0.001 | 1.68 (1.41-1.99) | <0.001 |
| Adult failure to thrive | 53 (1%) | 145 (0%) | 3.52 (2.57-4.81) | <0.001 | 2.14 (1.58-2.90) | <0.001 |
| Cachexia | 37 (1%) | 119 (0%) | 2.99 (2.07-4.32) | <0.001 | 1.85 (1.28-2.68) | 0.038 |
| Polyphagia | 33 (0%) | 111 (0%) | 2.86 (1.94-4.22) | <0.001 | 1.88 (1.27-2.77) | 0.06 |
| Adrenogenital disorders | 16 (0%) | 37 (0%) | 4.16 (2.32-7.48) | <0.001 | 2.36 (1.27-4.38) | 0.25 |
| **Infectious Disease** |  |  |  |  |  |  |
| Viral hepatitis | 140 (2%) | 534 (1%) | 2.52 (2.10-3.03) | <0.001 | 1.60 (1.34-1.92) | <0.001 |
| Other sexually transmitted infections (not HIV or hepatitis) | 105 (2%) | 460 (1%) | 2.20 (1.78-2.71) | <0.001 | 1.50 (1.21-1.86) | 0.008 |
| Human immunodeficiency virus [HIV] disease | 78 (1%) | 79 (0%) | 9.50 (6.96-12.98) | <0.001 | 6.42 (4.67-8.84) | <0.001 |
| **Circulatory System** |  |  |  |  |  |  |
| Cardiac conduction disorders | 652 (10%) | 2737 (4%) | 2.29 (2.11-2.49) | <0.001 | 1.43 (1.33-1.54) | <0.001 |
| Hypotension | 457 (7%) | 1844 (3%) | 2.38 (2.16-2.63) | <0.001 | 1.48 (1.35-1.62) | <0.001 |
| Orthostatic hypotension | 170 (3%) | 564 (1%) | 2.90 (2.45-3.44) | <0.001 | 1.85 (1.57-2.18) | <0.001 |
| Other cardiac conduction disorders | 121 (2%) | 264 (0%) | 4.41 (3.56-5.46) | <0.001 | 2.77 (2.25-3.42) | <0.001 |
| Iatrogenic hypotension | 63 (1%) | 157 (0%) | 3.86 (2.89-5.17) | <0.001 | 2.33 (1.74-3.12) | <0.001 |
| **Dermatologic** |  |  |  |  |  |  |
| Acne | 1028 (15%) | 8453 (13%) | 1.17 (1.10-1.24) | <0.001 | 0.80 (0.75-0.84) | <0.001 |
| Hirsutism | 86 (1%) | 284 (0%) | 2.91 (2.29-3.70) | <0.001 | 1.94 (1.52-2.48) | <0.001 |
| Diffuse diseases of connective tissue | 65 (1%) | 294 (0%) | 2.13 (1.63-2.78) | <0.001 | 1.37 (1.05-1.78) | 0.74 |
| Changes in skin texture | 36 (1%) | 164 (0%) | 2.11 (1.47-3.03) | <0.001 | 1.37 (0.96-1.96) | >0.99 |
| **Digestive System** |  |  |  |  |  |  |
| Symptoms involving digestive system | 1026 (15%) | 5897 (9%) | 1.67 (1.57-1.78) | <0.001 | 1.09 (1.04-1.16) | 0.053 |
| Irritable bowel syndrome | 280 (4%) | 1592 (2%) | 1.69 (1.49-1.92) | <0.001 | 1.09 (0.96-1.23) | >0.99 |
| Personal history of diseases of digestive system | 219 (3%) | 996 (2%) | 2.12 (1.83-2.44) | <0.001 | 1.35 (1.18-1.55) | <0.001 |
| **Genitourinary** |  |  |  |  |  |  |
| Menopausal and postmenopausal disorders | 1067 (16%) | 2001 (3%) | 5.13 (4.78-5.50) | <0.001 | 3.32 (3.07-3.60) | <0.001 |
| Need for hormone replacement therapy (postmenopausal) | 956 (14%) | 727 (1%) | 12.65 (11.53-13.89) | <0.001 | 8.15 (7.38-9.00) | <0.001 |
| Breast conditions, congenital or relating to hormones | 281 (4%) | 752 (1%) | 3.60 (3.14-4.11) | <0.001 | 2.28 (1.98-2.62) | <0.001 |
| Male infertility and abnormal spermatozoa | 155 (2%) | 455 (1%) | 3.28 (2.74-3.93) | <0.001 | 2.17 (1.79-2.63) | <0.001 |
| Urinary complications nec | 49 (1%) | 118 (0%) | 4.00 (2.87-5.57) | <0.001 | 2.27 (1.59-3.23) | <0.001 |
| **Hematopoietic** |  |  |  |  |  |  |
| Polycythemia, secondary | 94 (1%) | 229 (0%) | 3.95 (3.11-5.02) | <0.001 | 2.54 (1.98-3.25) | <0.001 |
| Abnormality of red blood cells | 78 (1%) | 240 (0%) | 3.13 (2.43-4.03) | <0.001 | 1.99 (1.53-2.58) | <0.001 |
| Deficiency anemias | 76 (1%) | 264 (0%) | 2.77 (2.15-3.57) | <0.001 | 1.67 (1.31-2.13) | <0.001 |
| **Neurological** |  |  |  |  |  |  |
| Sleep disorders | 2329 (35%) | 9609 (15%) | 2.33 (2.25-2.42) | <0.001 | 1.49 (1.44-1.55) | <0.001 |
| Insomnia | 1811 (27%) | 6183 (10%) | 2.82 (2.69-2.95) | <0.001 | 1.80 (1.72-1.88) | <0.001 |
| Chronic pain | 1136 (17%) | 4682(7%) | 2.33 (2.20-2.48) | <0.001 | 1.49 (1.41-1.57) | <0.001 |
| Restless legs syndrome | 168 (3%) | 686 (1%) | 2.36 (1.99-2.78) | <0.001 | 1.49 (1.26-1.75) | <0.001 |
| Migraine with aura | 269 (4%) | 1206 (2%) | 2.15 (1.89-2.44) | <0.001 | 1.36 (1.20-1.55) | <0.001 |
| Parasomnia | 224 (3%) | 370 (1%) | 5.83 (4.94-6.86) | <0.001 | 3.66 (3.11-4.32) | <0.001 |
| Sleep related movement disorders | 209 (3%) | 858 (1%) | 2.34 (2.02-2.72) | <0.001 | 1.48 (1.28-1.71) | <0.001 |
| Hypersomnia | 177 (3%) | 761 (1%) | 2.24 (1.90-2.63) | <0.001 | 1.45 (1.24-1.70) | <0.001 |

***Notes.*** CI, confidence interval. ^a^, p-value controlled for family-wise error rate (family of test = 38). ^b^, Adjusted for birth cohort, birth certificate sex, race and ethnicity, with the number of unique ICD codes as an offset.
