# Supplementary material for: Unveiling health disparities: Diagnostic prevalences in a transgender cohort versus matched controls
Source: PLoS One. 2025 Aug 6;20(8):e0329849. doi: 10.1371/journal.pone.0329849 (PMC12327606; doi:10.1371/journal.pone.0329849)
Supplement: S2 Table — Prevalence of mental health and neurodevelopmental clinical phenotypes for TGD cases and population controls, and association of phenotypes with TGD group membership. (DOCX) [file pone.0329849.s002.docx]

**S2 Table. Prevalence of mental health and neurodevelopmental clinical phenotypes for TGD cases and population controls, and association of phenotypes with TGD group membership**

| **Clinical Phenotypes** | **TGD Cases**  **(N=6664)** | **Population Controls (N=64124)** | **Prevalence Ratio**  **(PR)**  **(95% CI)** | ***p*-value^a^** | **Adjusted PR^b^**  **(95% CI)** | ***p*-value^a^** |
| --- | --- | --- | --- | --- | --- | --- |
| **Mental Health** |  |  |  |  |  |  |
| Mood disorders | 4696 (70%) | 15878 (25%) | 2.85 (2.79-2.91) | <0.001 | 1.79 (1.74-1.84) | <0.001 |
| Depression | 4553 (68%) | 15128 (24%) | 2.90 (2.83-2.96) | <0.001 | 1.82 (1.77-1.88) | <0.001 |
| Anxiety disorder | 4253 (64%) | 16650 (26%) | 2.46 (2.40-2.51) | <0.001 | 1.58 (1.54-1.62) | <0.001 |
| Other mental disorder | 3547 (53%) | 25841 (40%) | 1.32 (1.29-1.35) | <0.001 | 0.88 (0.86-0.91) | <0.001 |
| Suicidal ideation or attempt | 2438 (37%) | 4927 (8%) | 4.76 (4.57-4.96) | <0.001 | 3.05 (2.93-3.18) | <0.001 |
| Tobacco use disorder | 2152 (32%) | 8835 (14%) | 2.34 (2.25-2.44) | <0.001 | 1.47 (1.40-1.53) | <0.001 |
| Agorophobia, social phobia, and panic disorder | 1225 (18%) | 2707 (4%) | 4.35 (4.09-4.64) | <0.001 | 2.81 (2.64-2.99) | <0.001 |
| Non-alcohol substance use disorders (Substance addiction and disorders) | 1161 (17%) | 3739 (6%) | 2.99 (2.81-3.18) | <0.001 | 1.91 (1.80-2.03) | <0.001 |
| Posttraumatic stress disorder | 1154 (17%) | 1931 (3%) | 5.75 (5.37-6.16) | <0.001 | 3.73 (3.49-3.98) | <0.001 |
| Bipolar | 1122 (17%) | 2464 (4%) | 4.38 (4.10-4.68) | <0.001 | 2.82 (2.65-3.01) | <0.001 |
| Suicide or self-inflicted injury | 1104 (17%) | 2153 (3%) | 4.93 (4.61-5.28) | <0.001 | 3.15 (2.95-3.37) | <0.001 |
| Alcohol-related disorders | 911 (14%) | 2954 (5%) | 2.97 (2.77-3.18) | <0.001 | 1.95 (1.82-2.09) | <0.001 |
| Personality disorders | 730 (11%) | 999 (2%) | 7.03 (6.41-7.71) | <0.001 | 4.59 (4.19-5.03) | <0.001 |
| Dysthymic disorder | 681 (10%) | 2268 (4%) | 2.89 (2.66-3.14) | <0.001 | 1.91 (1.76-2.07) | <0.001 |
| Adjustment reaction | 658 (10%) | 2091 (3%) | 3.03 (2.78-3.29) | <0.001 | 2.03 (1.87-2.20) | <0.001 |
| Schizophrenia and other psychotic disorders | 631 (9%) | 1274 (2%) | 4.77 (4.35-5.23) | <0.001 | 3.08 (2.81-3.37) | <0.001 |
| Eating disorder | 342 (5%) | 568 (1%) | 5.79 (5.08-6.61) | <0.001 | 3.51 (3.07-4.01) | <0.001 |
| Anorexia nervosa | 110 (2%) | 156 (0%) | 6.79 (5.32-8.65) | <0.001 | 4.19 (3.27-5.35) | <0.001 |
| Obsessive-compulsive disorders | 335 (5%) | 902 (1%) | 3.57 (3.16-4.04) | <0.001 | 2.36 (2.08-2.67) | <0.001 |
| Psychogenic and somatoform disorders | 318 (5%) | 937 (1%) | 3.27 (2.88-3.70) | <0.001 | 2.11 (1.86-2.38) | <0.001 |
| Paranoid disorders | 232 (3%) | 410 (1%) | 5.44 (4.64-6.38) | <0.001 | 3.51 (3.00-4.11) | <0.001 |
| Acute reaction to stress | 203 (3%) | 846 (1%) | 2.31 (1.99-2.69) | <0.001 | 1.45 (1.25-1.68) | <0.001 |
| Tension headache | 174 (3%) | 1000 (2%) | 1.67 (1.43-1.96) | <0.001 | 1.09 (0.93-1.27) | >0.99 |
| Somatoform disorder | 167 (3%) | 447 (1%) | 3.60 (3.01-4.29) | <0.001 | 2.27 (1.90-2.70) | <0.001 |
| Dissociative disorder | 125 (2%) | 128 (0%) | 9.40 (7.35-12.01) | <0.001 | 5.92 (4.64-7.54) | <0.001 |
| Phobia | 109 (2%) | 222 (0%) | 4.72 (3.76-5.93) | <0.001 | 3.17 (2.51-3.99) | <0.001 |
| **Neurodevelopmental** |  |  |  |  |  |  |
| Attention-deficit hyperactivity disorder | 1398 (21%) | 5831 (9%) | 2.31 (2.19-2.43) | <0.001 | 1.61 (1.53-1.69) | <0.001 |
| Autism | 483 (7%) | 1040 (2%) | 4.47 (4.02-4.96) | <0.001 | 3.32 (2.99-3.68) | <0.001 |

***Notes.*** PR, prevalence ratio. CI, confidence interval. ^a^, p-value controlled for family-wise error rate (family of test = 28). ^b^, Adjusted for birth cohort, birth certificate sex, race and ethnicity with the number of unique ICD codes as an offset.
