# Supplementary material for: Unveiling health disparities: Diagnostic prevalences in a transgender cohort versus matched controls
Source: PLoS One. 2025 Aug 6;20(8):e0329849. doi: 10.1371/journal.pone.0329849 (PMC12327606; doi:10.1371/journal.pone.0329849)
Supplement: S3 Table — Diagnostic codes, procedural codes, and medication classes used as orthogonal evidence supporting TGD identity. (DOCX) [file pone.0329849.s003.docx]

**S3a Table. Diagnosis Codes**

| **Code Type** | **Code** | **Description** |
| --- | --- | --- |
| ICD-10 | F64.0 | [transsexualism] |
| ICD-10 | F64.1 | [gender identity disorder in adolescence and adulthood] |
| ICD-10 | F64.2 | [gender identity disorder of childhood |
| ICD-10 | F64.8 | [other gender identity disorders] |
| ICD-10 | F64.9 | [gender identity disorder, unspecified] |
| ICD-9 | 302.5 | [trans-sexualism with unspecified sexual history] |
| ICD-9 | 302.5 | [trans-sexualism with unspecified sexual history] |
| ICD-9 | 302.5 | [trans-sexualism with unspecified sexual history] |
| ICD-9 | 302.5 | [trans-sexualism with unspecified sexual history] |
| ICD-9 | 302.5 | [trans-sexualism with unspecified sexual history] |
| ICD-9 | 302.50 | [trans-sexualism with unspecified sexual history] |
| ICD-9 | 302.51 | [trans-sexualism with asexual history convert] |
| ICD-9 | 302.52 | [Trans-sexualism with homosexual history convert] |
| ICD-9 | 302.53 | [Trans-sexualism with heterosexual history convert 302.53] |
| ICD-9 | 302.6 | [gender identity disorder in children] |
| ICD-9 | 302.85 | [gender identity disorder in adolescents or adults] |

*Note.* We recognize that some of these codes reflect obsolete and potentially offensive terminology. We have elected to be comprehensive in the inclusion of codes used at various points and with differing language to capture TGD individuals, recognizing the limitations of several codes in their accuracy and sensitivity.

**S3b Table. Procedure Codes (CPT and ICD)**

| **CPT Codes** | |
| --- | --- |
| **CPT Code** | **Code Description** |
| 11920-11922 | Tattooing, intradermal introduction of insoluble opaque pigments to correct color defects of skin, including micropigmentation; 6.0 sq cm or less [when specified for nipple/areola reconstruction after breast surgery; includes codes 11920, 11921, 11922] |
| 17380 | Electrolysis epilation, each 30 minutes |
| 17999 | Unlisted procedure, skin, mucous membrane and subcutaneous tissue [when specified as permanent hair removal by laser] |
| 19303 | Mastectomy, simple, complete |
| 19318 | Reduction mammaplasty |
| 19325 | Mammaplasty, augmentation; with prosthetic implant |
| 19350 | Nipple/areola reconstruction |
| 54125 | Amputation of penis; complete |
| 54520 | Orchiectomy, simple (including subcapsular), with or without testicular prosthesis, scrotal or inguinal approach |
| 54660 | Insertion of testicular prosthesis |
| 54690 | Laparoscopy, surgical; orchiectomy |
| 55180 | Scrotoplasty; complicated |
| 56625 | Vulvectomy, simple; complete |
| 56800 | Plastic repair of introitus |
| 57110 | Vaginectomy, complete removal of vaginal wall; |
| 57291 | Construction of artificial vagina; without graft |
| 57292 | Construction of artificial vagina; with graft |
| 57295 | Revision (including removal) of prosthetic vaginal graft; vaginal approach |
| 57296 | Revision (including removal) of prosthetic vaginal graft; open abdominal approach |
| 57426 | Revision (including removal) of prosthetic vaginal graft, laparoscopic approach |
| 58150 | Total abdominal hysterectomy (corpus and cervix), with or without removal of tube(s), with or without removal of ovary(s); |
| 58552 | Laparoscopy, surgical, with vaginal hysterectomy, for uterus 250 g or less; with removal of tube(s) and/or ovary(s) |
| 58554 | Laparoscopy, surgical, with vaginal hysterectomy, for uterus greater than 250 g; with removal of tube(s) and/or ovary(s) |
| 58570 | Laparoscopy, surgical, with total hysterectomy, for uterus 250 g or less; |
| 58571 | Laparoscopy, surgical, with total hysterectomy, for uterus 250 g or less; with removal of tube(s) and/or ovary(s) |
| 58572 | Laparoscopy, surgical, with total hysterectomy, for uterus greater than 250 g; |
| 58573 | Laparoscopy, surgical, with total hysterectomy, for uterus greater than 250 g; with removal of tube(s) and/or ovary(s) |
| **ICD-10 Procedure Codes** | |
| **ICD Code** | **Code Description** |
| 0HBV0ZZ-0HBVXZZ | Excision of breast, bilateral [by approach; includes codes 0HBV0ZZ, 0HBV3ZZ, 0HBV7ZZ, 0HBV8ZZ, 0HBVXZZ] |
| 0HDSXZZ | Extraction of hair, external approach |
| 0HRW07Z-0HRXXKZ | Replacement of nipple [by approach; includes codes 0HRW07Z, 0HRW0JZ, 0HRW0KZ, 0HRW3JZ, 0HRW3KZ, 0HRW37Z, 0HRWX7Z, 0HRWXJZ, 0HRWXKZ, 0HRX07Z, 0HRX0JZ, 0HRX0KZ, 0HRX3JZ, 0HRX3KZ, 0HRX37Z, 0HRXX7Z, 0HRXXJZ, 0HRXXKZ] |
| 0UQG0ZZ | Repair vagina, open approach |
| 0UQJ0ZZ-0UQJXZZ | Repair clitoris [by approach; includes codes 0UQJ0ZZ, 0UQJXZZ] |
| 0UT20ZZ-0UT2FZZ | Resection of bilateral ovaries [by approach; includes codes 0UT20ZZ, 0UT24ZZ, 0UT27ZZ, 0UT28ZZ, 0UT2FZZ] |
| 0UT70ZZ-0UT7FZZ | Resection of bilateral fallopian tubes [by approach; includes codes 0UT70ZZ, 0UT74ZZ, 0UT77ZZ, 0UT78ZZ, 0UT7FZZ] |
| 0UT90ZZ-0UT9FZZ | Resection of uterus [by approach; includes codes 0UT90ZZ, 0UT94ZZ, 0UT97ZZ, 0UT98ZZ, 0UT9FZZ] |
| 0UTC0ZZ-0UTC8ZZ | Resection of cervix [by approach; includes codes 0UTC0ZZ, 0UTC4ZZ, 0UTC7ZZ, 0UTC8ZZ] |
| 0UTG0ZZ-0UTG8ZZ | Resection of vagina [by approach; includes codes 0UTG0ZZ, 0UTG4ZZ, 0UTG7ZZ, 0UTG8ZZ] |
| 0UTJ0ZZ-0UTJXZZ | Resection of clitoris [by approach; includes codes 0UTJ0ZZ, 0UTJXZZ] |
| 0UTM0ZZ-0UTMXZZ | Resection of vulva [by approach; includes codes 0UTM0ZZ, 0UTMXZZ] |
| 0VRC0JZ | Replacement of bilateral testes with synthetic substitute, open approach |
| 0VTC0ZZ-0VTC4ZZ | Resection of bilateral testes [by approach; includes codes 0VTC0ZZ, 0VTC4ZZ] |
| 0VTS0ZZ-0VTSXZZ | Resection of penis [by approach; includes codes 0VTS0ZZ, 0VTS4ZZ, 0VTSXZZ] |
| 0VUS07Z-0VUSX7Z | Supplement penis with autologous tissue substitute [by approach, includes codes 0VUS07Z, 0VUS47Z, 0VUSX7Z] |
| 0VUS0JZ-0VUSXJZ | Supplement penis with synthetic substitute [by approach; includes codes 0VUS0JZ, 0VUS4JZ, 0VUSXJZ] |
| 0VUS0KZ-0VUSXKZ | Supplement penis with nonautologous tissue substitute [by approach; includes codes 0VUS0KZ, 0VUS4KZ, 0VUSXKZ] |
| 0W4M070 | Creation of vagina in male perineum with autologous tissue substitute, open approach |
| 0W4M0J0 | Creation of vagina in male perineum with synthetic substitute, open approach |
| 0W4M0K0 | Creation of vagina in male perineum with nonautologous tissue substitute, open approach |
| 0W4M0Z0 | Creation of vagina in male perineum, open approach |
| 0W4N071 | Creation of penis in female perineum with autologous tissue substitute, open approach |
| 0W4N0J1 | Creation of penis in female perineum with synthetic substitute, open approach |
| 0W4N0K1 | Creation of penis in female perineum with nonautologous tissue substitute, open approach |
| 0W4N0Z1 | Creation of penis in female perineum, open approach |

*Note.* Where relevant, cases with a diagnosis of reproductive organ related cancer exists on same record as procedure were excluded.

**S3c Table. Prescription Drug Classes**

| Estrogen |
| --- |
| Contraceptive |
| Estradiol |
| Estrogens |
| Premarin |
| Androgens-anabolic |
| Testosterone |
| Leuprolide acetate |
| Testosterone |
| Conjugated estrogen |
| Histrelin acetate |
| Megestrol |
| Goserelin |

*Notes.* Drugs were searched for as imbedded in text (with wildcards before/after each listed term) as well as using the associated NDC codes. Prescriptions were cross matched with sex records to identify incongruence.
