# Supplementary material for: Unveiling health disparities: Diagnostic prevalences in a transgender cohort versus matched controls
Source: PLoS One. 2025 Aug 6;20(8):e0329849. doi: 10.1371/journal.pone.0329849 (PMC12327606; doi:10.1371/journal.pone.0329849)
Supplement: S4 Table — Corresponding PheWAS codes for medical and mental health/neurodevelopmental clinical phenotypes of interest. (DOCX) [file pone.0329849.s004.docx]

**S4a Table. Phenotypes and PHEWAS Coding for Medical Clinical Phenotypes**

| **Clinical Phenotypes** | **PHEWAS Coding** |
| --- | --- |
| **Endocrine/Metabolic** |  |
| Other endocrine disorders | Phecode 259 |
| Anorexia | Phecode 260.6 |
| Other nutritional deficiency | Phecode 263 |
| Adult failure to thrive | Phecode 260.3 |
| Cachexia | Phecode 260.1 |
| Polyphagia | Phecode 260.7 |
| Adrenogenital disorders | Phecode 255.3 |
| **Infectious Disease** |  |
| Viral hepatitis | Phecode 70 |
| Other sexually transmitted infections (not HIV or hepatitis) | Phecode 90 |
| Human immunodeficiency virus [HIV] disease | Phecode 71 |
| **Circulatory System** |  |
| Cardiac conduction disorders | Phecode 426 |
| Hypotension | Phecode 458 |
| Orthostatic hypotension | Phecode 458.1 |
| Other cardiac conduction disorders | Phecode 426.8 |
| Iatrogenic hypotension | Phecode 458.2 |
| **Dermatologic** |  |
| Acne | Phecode 706.1 |
| Hirsutism | Phecode 704.2 |
| Diffuse diseases of connective tissue | Phecode 709 |
| Changes in skin texture | Phecode 687.3 |
| **Digestive System** |  |
| Symptoms involving digestive system | Phecode 561 |
| Irritable bowel syndrome | Phecode 564.1 |
| Personal history of diseases of digestive system | Phecode 564.9 |
| **Genitournary** |  |
| Menopausal and postmenopausal disorders | Phecode 627 |
| Need for hormone replacement therapy (postmenopausal) | Phecode 627.22 |
| Breast conditions, congenital or relating to hormones | Phecode 612 |
| Male infertility and abnormal spermatozoa | Phecode 609 |
| Urinary complications nec | Phecode 597.2 |
| **Hematopoietic** |  |
| Polycythemia, secondary | Phecode 289.8 |
| Abnormality of red blood cells | Phecode 289.9 |
| Deficiency anemias | Phecode 281.9 |
| **Neurological** |  |
| Sleep disorders | Phecode 327 |
| Insomnia | Phecode 327.4 |
| Chronic pain | Phecode 338.2 |
| Migraine with aura | Phecode 340.1 |
| Parasomnia | Phecode 327.5 |
| Sleep related movement disorders | Phecode 327.7 |
| Hypersomnia | Phecode 327.1 |
| Restless legs syndrome | Phecode 327.71 |

**S4b Table. Phenotypes and Coding Origin for Clinical Phenotypes (Mental Health & Neurodevelopmental)**

| **Clinical Phenotypes** | **Coding Categorization** |
| --- | --- |
| **Mental Health** |  |
| Mood disorders | Phecode 296 |
| Depression | Phecode 296.2 |
|  |  |
| Anxiety disorder | Phecode 300.1 |
| Other mental disorder | Phecode 306 |
| Suicidal ideation or attempt | Phecode 297 |
| Tobacco use disorder | Phecode 318 |
| Agorophobia, social phobia, and panic disorder | Phecode 300.12 |
| Non-alcohol substance use disorders (Substance addiction and disorders) | Phecode 316 |
| Posttraumatic stress disorder | Phecode 300.9 |
| Bipolar | Phecode 296.1 |
| Suicide or self-inflicted injury | Phecode 297.2 |
| Alcohol-related disorders | Phecode 317 |
| Personality disorders | Phecode 301 |
| Dysthymic disorder | Phecode 300.4 |
| Adjustment reaction | Phecode 304 |
| Schizophrenia and other psychotic disorders | Phecode 295 |
| Antisocial/borderline personality disorder | Phecode 301.2 |
| Psychosis | Phecode 295.3 |
| Eating disorder | Phecode 305.2 |
| Obsessive-compulsive disorders | Phecode 300.3 |
| Psychogenic and somatoform disorders | Phecode 303 |
| Schizophrenia | Phecode 295.1 |
| Paranoid disorders | Phecode 295.2 |
| Acute reaction to stress | Phecode 300.8 |
| Tension headache | Phecode 306.9 |
| Somatoform disorder | Phecode 303.4 |
| Dissociative disorder | Phecode 303.1 |
| Anorexia nervosa | Phecode 305.21 |
| Phobia | Phecode 300.13 |
|  |  |
|  |  |
| **Neurodevelopmental** |  |
| Attention deficit hyperactivity disorder | Phecode 313.1 |
| Autism | Phecode 313.3 |
