# Supplementary material for: Unveiling health disparities: Diagnostic prevalences in a transgender cohort versus matched controls
Source: PLoS One. 2025 Aug 6;20(8):e0329849. doi: 10.1371/journal.pone.0329849 (PMC12327606; doi:10.1371/journal.pone.0329849)
Supplement: S5 Table — Demographic characteristics, medical clinical phenotypes, and mental health/neurodevelopmental clinical phenotypes subset by gender characteristic (transmasculine, transfeminine). (DOCX) [file pone.0329849.s005.docx]

**S5a Table. Prevalence of demographic characteristics for transmasculine and transfeminine gender characteristic groups in TGD cohort, with group differences**

| **Characteristics** | **Transfeminine (N=2638)** | **Transmasculine (N=3222)** | ***p*-value** |
| --- | --- | --- | --- |
| **Birth Certificate Sex** |  |  | <0.001^c^ |
| Male | 1160(44%) | 37(1%) | - |
| Female | 79(3%) | 1455(45%) | - |
| Unknown/Missing | 1399(53%) | 1730(54%) | - |
| **Birth Cohort** |  |  | <0.001^c^ |
| 1920s^a^ & 1930s^a^ | 41(2%) | 27(1%) | - |
| 1940s | 75(3%) | 42(1%) | - |
| 1950s | 153(6%) | 68(2%) | - |
| 1960s | 185(7%) | 94(3%) | - |
| 1970s | 208(8%) | 116(4%) | - |
| 1980s | 412(16%) | 312(10%) | - |
| 1990s | 787(30%) | 989(31%) | - |
| 2000s^a^ & 2010s^a^ | 504(19%) | 1218(38%) | - |
| Missing | 273(10%) | 356(11%) | - |
| **Race** |  |  | 0.014^c^ |
| American Indian or Alaska Native^a^ &  Native Hawaiian or Other Pacific  Islander^a^ | 25(1%) | 20(1%) | - |
| Asian | 23(1%) | 23(1%) | - |
| Black or African American | 21(1%) | 44(1%) | - |
| White | 2080(79%) | 2450(76%) | - |
| Multiple Races | 105(4%) | 133(4%) | - |
| Unknown/Missing | 384(15%) | 552(17%) | - |
| **Ethnicity** |  |  | 0.008^c^ |
| Hispanic | 304(12%) | 457(14%) | - |
| Non-Hispanic | 1866(71%) | 2185(68%) | - |
| Unknown | 468(18%) | 580(18%) | - |

***Notes.*** ^a^Categories aggregated due to small cell sizes in accordance with data use agreements. ^c^Chi-squared test

**S5b Table. Prevalence of medical clinical phenotypes in the TGD cohort by transmasculine and transfeminine gender characteristics, and association of phenotypes with gender characteristics**

| **Clinical Phenotypes** | **Transfeminine**  **(N=2638)** | **Transmasculine**  **(N=3222)** | **Prevalence Ratio**  **(PR)**  **(95% CI)** | ***p*-value^a^** | **Adjusted PR^b^**  **(95% CI)** | ***p*-value^a^** |
| --- | --- | --- | --- | --- | --- | --- |
| **Endocrine/Metabolic** |  |  |  |  |  |  |
| Other endocrine disorders | 492 (19%) | 532 (17%) | 0.89 (0.79-0.99) | >0.99 | 0.94 (0.81-1.10) | >0.99 |
| Anorexia | 89 (3%) | 98 (3%) | 0.90 (0.68-1.20) | >0.99 | 0.80 (0.56-1.15) | >0.99 |
| Other nutritional deficiency | 64 (2%) | 64 (2%) | 0.82 (0.58-1.15) | >0.99 | 1.21 (0.79-1.87) | >0.99 |
| Adult failure to thrive | 31 (1%) | 20 (1%) | 0.53 (0.30-0.92) | 0.97 | 0.97 (0.54-1.73) | >0.99 |
| Cachexia | 22 (1%) | 12 (0%) | 0.45 (0.22-0.90) | 0.92 | 0.86 (0.40-1.86) | >0.99 |
| Polyphagia | 17 (1%) | 11 (0%) | 0.53 (0.25-1.13) | >0.99 | 0.36 (0.11-1.11) | >0.99 |
| Adrenogenital disorders | <11^c^ | 12 (0%) | 3.28 (0.93-11.59) | >0.99 | 1.50 (0.23-9.65) | >0.99 |
| **Infectious Disease** |  |  |  |  |  |  |
| Viral hepatitis | 87 (3%) | 37 (1%) | 0.35 (0.24-0.51) | <0.001 | 0.44 (0.28-0.70) | 0.019 |
| Other sexually transmitted infections (not HIV or hepatitis) | 57 (2%) | 34 (1%) | 0.49 (0.32-0.74) | 0.034 | 0.58 (0.35-0.95) | >0.99 |
| Human immunodeficiency virus [HIV] disease | 59 (2%) | <11^c^ | 0.14 (0.07-0.27) | <0.001 | 0.18 (0.09-0.40) | <0.001 |
| **Circulatory System** |  |  |  |  |  |  |
| Cardiac conduction disorders | 332 (13%) | 234 (7%) | 0.58 (0.49-0.68) | <0.001 | 0.79 (0.67-0.93) | 0.21 |
| Hypotension | 209 (8%) | 191 (6%) | 0.75 (0.62-0.90) | 0.10 | 1.01 (0.83-1.23) | >0.99 |
| Orthostatic hypotension | 61 (2%) | 82 (3%) | 1.10 (0.79-1.53) | >0.99 | 1.51 (1.03-2.23) | >0.99 |
| Other cardiac conduction disorders | 55 (2%) | 52 (2%) | 0.77 (0.53-1.13) | >0.99 | 0.92 (0.59-1.45) | >0.99 |
| Iatrogenic hypotension | 32 (1%) | 23 (1%) | 0.59 (0.35-1.00) | >0.99 | 0.95 (0.53-1.72) | >0.99 |
| **Dermatologic** |  |  |  |  |  | >0.99 |
| Acne | 318 (12%) | 571 (18%) | 1.47 (1.29-1.67) | <0.001 | 1.15 (0.96-1.38) | >0.99 |
| Hirsutism | 26 (1%) | 46 (1%) | 1.45 (0.90-2.34) | >0.99 | 1.03 (0.57-1.86) | >0.99 |
| Diffuse diseases of connective tissue | 23 (1%) | 32 (1%) | 1.14 (0.67-1.94) | >0.99 | 1.88 (1.00-3.54) | >0.99 |
| Changes in skin texture | 13 (0%) | 18 (1%) | 1.13 (0.56-2.31) | >0.99 | 1.49 (0.67-3.29) | >0.99 |
| **Digestive System** |  |  |  | >0.99 |  | >0.99 |
| Symptoms involving digestive system | 418 (16%) | 453 (14%) | 0.89 (0.78-1.00) | >0.99 | 0.93 (0.81-1.07) | >0.99 |
| Irritable bowel syndrome | 104 (4%) | 134 (4%) | 1.05 (0.82-1.36) | >0.99 | 0.99 (0.72-1.37) | >0.99 |
| Personal history of diseases of digestive system | 107 (4%) | 79 (2%) | 0.60 (0.45-0.80) | 0.023 | 0.87 (0.62-1.23) | >0.99 |
| **Genitourinary** |  |  |  |  |  |  |
| Menopausal and postmenopausal disorders | 517 (20%) | 466 (14%) | 0.74 (0.66-0.83) | <0.001 | 0.80 (0.69-0.93) | 0.13 |
| Need for hormone replacement therapy (postmenopausal) | 483 (18%) | 399 (12%) | 0.68 (0.60-0.76) | <0.001 | 0.73 (0.62-0.86) | 0.008 |
| Breast conditions, congenital or relating to hormones | 141 (5%) | 115 (4%) | 0.67 (0.52-0.85) | 0.038 | 0.71 (0.52-0.98) | >0.99 |
| Male infertility and abnormal spermatozoa | 138 (5%) | <11^c^ | 0.03 (0.01-0.07) | <0.001 | 0.07 (0.02-0.17) | <0.001 |
| Urinary complications nec | 29 (1%) | 18 (1%) | 0.51 (0.28-0.91) | 0.89 | 0.70 (0.31-1.58) | >0.99 |
| **Hematopoietic** |  |  |  |  |  |  |
| Polycythemia, secondary | 20 (1%) | 65 (2%) | 2.66 (1.62-4.38) | 0.004 | 3.20 (1.67-6.13) | 0.015 |
| Abnormality of red blood cells | 18 (1%) | 49 (2%) | 2.23 (1.30-3.82) | 0.13 | 2.69 (1.49-4.88) | 0.042 |
| Deficiency anemias | 43 (2%) | 27 (1%) | 0.51 (0.32-0.83) | 0.24 | 0.94 (0.57-1.55) | >0.99 |
| **Neurological** |  |  |  |  |  |  |
| Sleep disorders | 909 (34%) | 1064 (33%) | 0.96 (0.89-1.03) | >0.99 | 0.94 (0.86-1.03) | >0.99 |
| Insomnia | 699 (26%) | 818 (25%) | 0.96 (0.88-1.05) | >0.99 | 0.92 (0.82-1.03) | >0.99 |
| Chronic pain | 442 (17%) | 517 (16%) | 0.96 (0.85-1.08) | >0.99 | 1.16 (1.02-1.31) | 0.90 |
| Restless legs syndrome | 80 (3%) | 67 (2%) | 0.69 (0.50-0.94) | 0.80 | 1.03 (0.69-1.54) | >0.99 |
| Migraine with aura | 104 (4%) | 121 (4%) | 0.95 (0.74-1.23) | >0.99 | 0.82 (0.59-1.14) | >0.99 |
| Parasomnia | 77 (3%) | 100 (3%) | 1.06 (0.79-1.42) | >0.99 | 0.84 (0.56-1.25) | >0.99 |
| Sleep related movement disorders | 96 (4%) | 87 (3%) | 0.74 (0.56-0.99) | >0.99 | 1.10 (0.78-1.57) | >0.99 |
| Hypersomnia | 72 (3%) | 82 (3%) | 0.93 (0.68-1.27) | >0.99 | 1.35 (0.91-2.01) | >0.99 |

***Notes.*** CI, confidence interval. ^a^, p-value controlled for family-wise error rate (family of test = 38). ^b^, Adjusted for birth cohort, birth certificate sex, race and ethnicity with the number of unique ICD codes as an offset. ^c^, Cell sizes <11 suppressed in accordance with data use agreements.

**S5c Table. Prevalence of mental health and neurodevelopmental clinical phenotypes in the TGD cohort by transmasculine and transfeminine gender characteristics, and association of phenotypes with gender characteristics**

| **Clinical Phenotypes** | **Transfeminine**  **(N=2638)** | **Transmasculine**  **(N=3222)** | **Prevalence Ratio**  **(PR)**  **(95% CI)** | ***p*-value^a^** | **Adjusted PR^b^**  **(95% CI)** | ***p*-value^a^** |
| --- | --- | --- | --- | --- | --- | --- |
| **Mental Health** |  |  |  |  |  |  |
| Mood disorders | 1796(68%) | 2252(70%) | 1.03 (0.99-1.06) | >0.99 | 0.90 (0.84-0.97) | 0.17 |
| Depression | 1733(66%) | 2193(68%) | 1.04 (1.00-1.07) | >0.99 | 0.91 (0.84-0.98) | 0.28 |
| Anxiety disorder | 1610(61%) | 2048(64%) | 1.04 (1.00-1.08) | >0.99 | 0.91 (0.85-0.98) | 0.48 |
| Other mental disorder | 1380(52%) | 1671(52%) | 0.99 (0.94-1.04) | >0.99 | 0.92 (0.85-0.99) | 0.59 |
| Suicidal ideation or attempt | 854(32%) | 1170(36%) | 1.12 (1.04-1.21) | 0.048 | 0.89 (0.80-0.98) | 0.68 |
| Tobacco use disorder | 884(34%) | 1004(31%) | 0.93 (0.86-1.00) | >0.99 | 1.02 (0.92-1.14) | >0.99 |
| Agorophobia, social phobia, and panic disorder | 421(16%) | 614(19%) | 1.19 (1.07-1.34) | 0.06 | 1.00 (0.85-1.17) | >0.99 |
| Non-alcohol substance use disorders | 483(18%) | 495(15%) | 0.84 (0.75-0.94) | 0.07 | 0.89 (0.77-1.03) | >0.99 |
| Posttraumatic stress disorder | 369(14%) | 568(18%) | 1.26 (1.12-1.42) | 0.006 | 1.01 (0.87-1.17) | >0.99 |
| Bipolar | 409(16%) | 529(16%) | 1.06 (0.94-1.19) | >0.99 | 1.06 (0.90-1.25) | >0.99 |
| Suicide or self-inflicted injury | 361(14%) | 567(18%) | 1.29 (1.14-1.45) | <0.001 | 1.08 (0.91-1.27) | >0.99 |
| Alcohol-related disorders | 391(15%) | 369(11%) | 0.77 (0.68-0.88) | 0.003 | 0.88 (0.74-1.04) | >0.99 |
| Personality disorders | 278(11%) | 331(10%) | 0.97 (0.84-1.13) | >0.99 | 0.90 (0.75-1.09) | >0.99 |
| Dysthymic disorder | 283(11%) | 317(10%) | 0.92 (0.79-1.07) | >0.99 | 0.94 (0.77-1.15) | >0.99 |
| Adjustment reaction | 255(10%) | 323(10%) | 1.04 (0.89-1.21) | >0.99 | 1.09 (0.90-1.33) | >0.99 |
| Schizophrenia and other psychotic disorders | 286(11%) | 254(8%) | 0.73 (0.62-0.85) | 0.003 | 0.85 (0.69-1.05) | >0.99 |
| Anorexia nervosa | 96(4%) | 185(6%) | 1.58 (1.24-2.01) | 0.006 | 0.97 (0.68-1.39) | >0.99 |
| Eating disorder | 26(1%) | 66(2%) | 2.08 (1.32-3.26) | 0.042 | 1.08 (0.57-2.06) | >0.99 |
| Obsessive-compulsive disorders | 127(5%) | 149(5%) | 0.96 (0.76-1.21) | >0.99 | 0.77 (0.56-1.06) | >0.99 |
| Psychogenic and somatoform disorders | 120(5%) | 161(5%) | 1.10 (0.87-1.38) | >0.99 | 1.09 (0.82-1.44) | >0.99 |
| Paranoid disorders | 103(4%) | 91(3%) | 0.72 (0.55-0.95) | 0.62 | 0.80 (0.55-1.15) | >0.99 |
| Acute reaction to stress | 81(3%) | 93(3%) | 0.94 (0.70-1.26) | >0.99 | 0.93 (0.62-1.42) | >0.99 |
| Tension headache | 50(2%) | 96(3%) | 1.57 (1.12-2.20) | 0.24 | 1.67 (1.05-2.65) | 0.87 |
| Somatoform disorder | 63(2%) | 84(3%) | 1.09 (0.79-1.51) | >0.99 | 1.02 (0.66-1.56) | >0.99 |
| Dissociative disorder | 42(2%) | 66(2%) | 1.29 (0.88-1.89) | >0.99 | 1.29 (0.79-2.11) | >0.99 |
| Phobia | 15(1%) | 81(3%) | 4.42 (2.55-7.65) | <0.001 | 4.47 (2.36-8.45) | <0.001 |
| **Neurodevelopmental** |  |  |  |  |  |  |
| Attention-deficit hyperactivity disorder | 557(21%) | 613(19%) | 0.90 (0.81-1.00) | >0.99 | 0.73 (0.63-0.84) | <0.001 |
| Autism | 230(9%) | 180(6%) | 0.64 (0.53-0.77) | <0.001 | 0.48 (0.37-0.63) | <0.001 |

***Notes.*** PR, prevalence ratio. CI, confidence interval. ^a^, p-value controlled for family-wise error rate (family of test = 28). ^b^, Adjusted for birth cohort, birth certificate sex, race and ethnicity with the number of unique ICD codes as an offset.
