# Supplementary material for: Unveiling health disparities: Diagnostic prevalences in a transgender cohort versus matched controls
Source: PLoS One. 2025 Aug 6;20(8):e0329849. doi: 10.1371/journal.pone.0329849 (PMC12327606; doi:10.1371/journal.pone.0329849)
Supplement: Attachment — Submitted filename: Response to Reviewers.docx [file pone.0329849.s006.docx]

Dear Dr. Turban,

Thank you for considering our manuscript PONE-D-25-11456 “Unveiling Health Disparities: Diagnostic Prevalences in a Transgender Cohort versus Matched Controls”.

We thank the reviewers for their thoughtful feedback. We are pleased to provide you with a point-by-point response to the reviewer’s comments as well as an updated manuscript with edits highlighted using track changes.

Summary of key additions:

- Strengthened introduction to contextualize our study in a more descriptive scaffold of current literature
- Provided methodological clarifications, particularly regarding ICD codes and disclaimers made in our previous text, without sufficient explanation
- Added additional discussion points as guided by reviewer feedback, to place our findings more concretely in the current context of the field and American landscape

An exhaustive description of changes is contained in the individual responses to reviewers. Please let us know if there are any other recommended changes or additional questions.

Point by point responses **(replies in blue bold text)**

### Journal Requirements and Editor Comments

*Journal Requirements*

Please ensure that your manuscript meets PLOS ONE's style requirements, including those for file naming.

**We have ensured our manuscript meets style requirements.**

If your data cannot be made publicly available for ethical or legal reasons (e.g., public availability would compromise patient privacy), please explain your reasons on resubmission and your exemption request will be escalated for approval.

**Under the usage agreement with the Utah Population Database (UPDB), we are not permitted to provide publicly available data. UPDB data is not publicly available to protect individual privacy, ensure ethical research practices, and maintain data security. Access is granted on a project-specific basis after thorough review and approval by the Resource for Genetic and Epidemiologic Research (RGE) and IRB. We would also like to note that TGD individuals are especially vulnerable given the current political climate, and the risk of identification warrants additional caution that underscores the necessity of abiding by the policies of UPDB data privacy.**

Please include a separate caption for each figure in your manuscript.

**We have included a separate caption for each figure.**

*Editor comments*

The introduction sets up that past research is based on convenience samples. The data presented also seem to be from a convenience sample. Can you clarify if this is the case?

**We apologize for any confusion here. We realize that the term “convenience” may be not adequately descriptive. We have reviewed all instances of that term and provided more specific language regarding our intent. We have also provided more explanation about the strengths and limitations of our approach compared with existing literature.**

While critiquing past studies for being small, it’s important to note that this sample if 6,664 TGD individuals is smaller than other studies (e.g., USTS).

**As mentioned above, we endeavored to clarify the context of our study in the broader trans health literature in an improved introduction, including a brief description of the USTS.**

It’s worth mentioning in the intro that TransPOP exists, as this is truly a probability sample (though ended up being small for this reason).

**We have included a brief mention of TransPOP in our introduction to contextualize this study among significant TGD studies.**

It’s important to acknowledge that different datasets have different strengths and weaknesses and that non-probability samples are essential for research on smaller populations (see Turban et al Transgender Health 2023 for a more detailed discussion on this).

**We hope that our improved introduction (including a citation to the above reference) captures the strengths of these studies while still introducing the unique capabilities of our dataset.**

Please comment on the choice to use some unusual diagnostic codes (e.g., F64.1 dual role transvestism — is this consistent with a TGD population?), as reviewer 1 also brought up.

**Thank you for giving us the opportunity to clarify our intent; we have responded to this point in response 2 to reviewer 1.**

### Reviewer 1:

1. Please define Phecodes early on (they are mentioned in the abstract). These are useful terms that I (and many readers, I suspect) are not familiar with. The “hierarchical Phecode system” is currently not mentioned until page 12.

**Great catch! We have included the following sentence in the last paragraph of our Introduction, so as to introduce the concept prior to the details of the methods section:**

**“We leverage Phecodes, collections of ICD codes intended to capture clinically meaningful concepts for research by corresponding to specific disease phenotypes, in order to assess distinct diagnostic umbrellas.”**

2. The umbrella term of gender diverse includes many ICD-10 codes that are obsolete (or even offensive to some). A note of contextualization about “transexual” codes would be welcome, even if as a footnote to the relevant table.

**This is an excellent point and suggestion, and is an opportunity to reiterate that the medicalization/pathologization of gender diversity leads to flawed and imprecise methods of categorizing TGD individuals and their experiences. The clinical vocabulary for TGD population is evolving, just as the vocabulary of society is evolving. We have included all possible ICD-10 codes, recognizing that some may be obsolete or offensive, to allow for a more accurate identification of cases in the population. For example, in 2016 the ICD-10 code F64.1 was used for “gender identity disorder in adolescents and adults”. This changed in 2017 when the codes were updated to add F64.0 (transsexualism) and change F64.1 to “dual role transvestism”.(ref: https://www.aapc.com/blog/36829-identify-transgender-coding-mishaps/?srsltid=AfmBOopKp75QVHWCMMaDJATA_ebDOeRX45irmbWkSNfN5YXI7_q2OipD) In 2025 Centers for Medicare and Medicaid Services (CMS) changed “gender dysphoria” to “sexual identity dysphoria”.(ref: https://www.cms.gov/medicare-coverage-database/view/article.aspx?articleid=53793) And the release of ICD11 re-classifies gender identity disorder of children (categorized as a Mental and Behavioral Disorder) to “gender incongruence of childhood [or adolescence and adulthood]” (categorized in “Conditions related to sexual health”). (ref:** [**https://www.who.int/standards/classifications/frequently-asked-questions/gender-incongruence-and-transgender-health-in-the-icd**](https://www.who.int/standards/classifications/frequently-asked-questions/gender-incongruence-and-transgender-health-in-the-icd)**).**

**We have added the following note to the Supplementary table of ICD codes: “Note. We recognize that some of these codes reflect obsolete and potentially offensive terminology. We have elected to be comprehensive in the inclusion of codes used at various points and with differing language to capture TGD individuals, recognizing the limitations of several codes in their accuracy and sensitivity.”**

3. On a related note: do all the codes add up to a true umbrella of “transgender and gender diverse (TGD)”? I think so, but am not sure. As TGD seems to be the more commonly used term these days, I would clarify the issue, and noting relevant shortcomings, if any. Stated alternatively: does ICD-10’s “gender incongruence” map neatly onto the generally accepted definition of TGD?

**We have added additional context and disclaimer, such as this section in the introduction “Medical record-based analyses come with their own limitations, such as necessitating that TGD individuals have accessed healthcare, have disclosed their identity to healthcare providers, and that their providers have documented their TGD identity and health needs in ways that can be captured. Nonetheless, they offer the potential to draw valuable conclusions about TGD populations meeting these criteria.”**

**The challenges and inadequacies of ascertaining TGD individuals from EHR data are multifold, especially considering the TGD individuals who will not seek gender-affirming care through any medical system, let alone in a way that is captured by the UPDB. While our data may not be a “true umbrella” (fully representative) of all TGD individuals, our inspection and curation have led us to the conclusion that this cohort is comprised of TGD individuals, leading to our subsequent analyses and interpretations.**

4. Suicide and suicide-related behavior is a major finding, and one with arguably the highest public health burden. I was surprised not to see it addressed in the discussion and think it is imperative to do so. Even if the findings are not necessarily new, they are important replications, especially in light of the study’s methodological rigor and sample size.

**Thank you for this point. You are correct in your assessment: as these findings are similar to previous research on suicidality in the TGD community, we did not emphasize them, but that is a substantial oversight on our part, particularly given the public health burden. We have added additional commentary to emphasize this point in the discussion.**

5. How does your statement of “an anticipated doubling of the size of the TGD population” resonate with the current political landscape and rhetoric? Although this may be speculative / editorial territory, it could be important to signpost it. For example, will some TGD individuals be more reluctant to seek care, to self-label, etc.? Could the TGD population actually contract over the coming years?

**We have endeavored to “signpost” the dangers of the current political landscape in our discussion as part of the “Broader implications” section, including the following point:**

**“As the impact of political and legislative forces on LGBTQ+ individuals’ mental and physical health in the US is a known phenomenon, these trends within TGD communities may be further exacerbated in the coming years.(Schlehofer et al. 2023) While societal and interpersonal pressures on the TGD community may lead to the avoidance of care or lower self-disclosure, such that the anticipated growth of the TGD community is obscured, this does not negate the evolving understanding of TGD patients’ unique and urgent healthcare needs.**

### Reviewer 2:

In the introduction, the authors cite self-report as a notable limitation of prior research on transgender health. The bigger issue is the sampling methodology. Self-report is a universal limitation of all survey research, and if one is to elevate it to point of an Achilles heel, then it renders all survey research suspect. The evidence they cite to bolster this point was based more in sampling and response bias than recall bias. The authors should focus their critique on the sampling methodologies.

**Thank you for raising this important point. We have provided more discussion about sampling methodologies, and also highlighted the strengths of survey research. We believe that our overview of existing literature and what our study contributes is more balanced now. Our wish is not to diminish the significance and strengths of survey research—particularly given the power of self-report and participant autonomy in trans health research—but to emphasize what our study adds to the literature.**

In the methods, the authors write “It is challenging to give an estimate of how many individuals are within the UPDB, as it contains millions of records derived from various sources.” In the results they write, “Estimating a TGD population prevalence was outside the scope of this study, as the research team did not have access to the relevant denominators…” These statements raise concerns about ascertainment of unique cases (i.e., uniques) in the dataset. Although the UPDB pulls data across multiple systems, it must do so on a unique case basis, which means that a denominator of uniques (i.e., universe) should be known. If uniques cannot be ascertained, then that is a major concern because people’s visits may be counted more than once. The authors need to clearly explain this issue of uniques in the Methods and explain why, if uniques were able to be identified, a denominator could not be ascertained.

**Thank you very much for raising this important point and allowing us to clarify. The UPDB does work on a unique case basis, but for privacy reasons it conducts linkages specific to each study only linking the necessary data. It is indeed possible to generate denominators, however, this was not data that we requested approval for in this study. We have removed the first statement quoted and within the second statement we added the word “permission” to clarify that it is a permissions-based issue rather than a concerning aspect of the data. TGD population prevalence was not one of our research aims, but we added this statement because our peers reviewing drafts of the manuscript were often curious about the prevalence, so we felt an explanation was warranted. We have also added a new citation which provides a detailed description of the UPDB (https://doi.org/10.51964/hlcs11681).**

The authors explain that “Administrative sex was missing or unknown for 3509

(53%) of TGD individuals and 34925 (54%) of the control cohort, indicating a consistent lack of documentation across cohorts.” Assumed consistency notwithstanding, and although this may be more understandable for TGD people for whom administrative data is fraught, it is less clear why more than half of assumedly cisgender people were missing administrative sex data. The authors should explain this lack of very basic data in EHR or why sex wasn’t available from vital records.

**Thank you for pointing this out. We now realize that our terminology was unclear. We used only Birth Certificate sex for consistency. Further, we have now explained in the Results that the consistency in missing or unknown administrative sex across both groups strongly reflects individuals born out of state, which was incorporated into the matching process (time living in the state). Despite availability, we elected not to use other available vital record or EHR data in this study since sometimes sex data collection reflects gender and sometimes it does not. We observe mixed M and F designations across multiple sources. Therefore, we chose to provide one consistent source of data to report from.**

The authors control for the number of ICD codes as a way of getting at informed presence bias, but the number of ICD codes does not account for disease severity and comorbidity. Because they have access to ICD diagnosis data, the authors may want to use a weighted comorbidity index score (e.g., weighted Elixhauser or Charlson), which is better at accounting for the severity of diagnosed conditions than a simple count.

**Thank you to the reviewer for inquiring about potentially adjusting for disease severity and comorbidity by including an Elixhauser or Charlson co-morbidity score. Our specific intent was to adjust for informative presence bias, not comorbidity/severity. Informative presence bias is a type of bias that arises when data collection in healthcare settings (like EHRs) is not random but rather reflects the presence of a patient with a particular condition. Informative presence bias can affect how data from EHRs is interpreted, potentially leading to inaccurate conclusions, hence we wanted to try to minimize informative presence bias in our study. Comorbidity indices such as the Elixhauser or Charlson are important predictors of outcomes like mortality and resource use (like length of stay). However, some of the co-occurring conditions that we examined in this study are included in these two indices and therefore including one of these indices might introduce another type of unintended bias into our study. Given the distinction between informative presence bias and the purpose of indices such as the Elixhauser, we hesitate to adjust our models based on comorbidity severity.**

The authors write: “Both “schizophrenia and other psychotic disorders” and “psychosis” were significantly elevated in the transfeminine cohort compared to the transmasculine, which may suggest additional marginalization either through biomedical stressors or diagnostic biases.” This sentence needs more explanation because it is unclear what kind of biomedical stressors or diagnostic biases that would specifically target transfeminine individuals and not transmasculine individuals or cisgender women.

**Thank you for the opportunity to clarify; we have added the following sentence with a pertinent citation and description of transmisogyny. We hope that this adds some clarity to the potential dynamics that may worsen health outcomes for transfeminine individuals, although further research is still needed.**

**“Psychological distress and suicidality have been reported to be higher in transfeminine compared to transmasculine young adults, attributed to structural oppression related to transmisogyny, which may lead to increased weathering in this group.(Wang et al. 2024) Research on manifestations of transmisogyny, or misogyny specific to transfeminine individuals, has discussed the risk of physical and verbal harassment and violence as well as social rejection in cisgender and transgender spaces specific to transfeminine individuals, which may in turn lead to an increase in stress-exacerbated conditions such as psychosis.”**

Minor

The attribution of the quote “If you’re not counted, then you don’t count” should be attributed to Judy Bradford’s research legacy, which predates the citation given. See: Jaffe, S. (2016). Judith Bradford: a pioneer of research on LGBT health. The Lancet, 387(10023), 1048.

**The authors are sincerely grateful for this note and the opportunity to update our citation and give explicit credit to the research legacy of Judith Bradford.**

***“The adage has been used for research assessing LGBT health disparities by field leaders such as Judith Bradford: “If you’re not counted, then you don’t count.”***

An additional limitation is potential misclassification because gender identity (transmasculine and transfeminine) were assumed by the research team based on other data in the patients’ records. This is triangulation, but it is not the gold standard of self-reported gender identity.

**We have added the following sentence to our discussion of limitations:**

**“We additionally inferred gender characteristics rather than using the gold standard of self-reported gender identity, which may lead to misclassification or oversimplification of patient identities.”**

### Reviewer 3:

1. In your statement on page 12 it says, “It is challenging to give an estimate of how many individuals are within the UPDB, as it contains millions of records derived from various sources.” Can you give examples or further define “various sources”?

**As noted above in response to Reviewer 2, we have made edits to the paper to be more clear about why we did not conduct a TGD population prevalence estimate. We also added a new citation which has details about UPDB and the various data sources:** [**https://doi.org/10.51964/hlcs11681**](https://doi.org/10.51964/hlcs11681)

2. Please write out PheWAS acronym the first time it is introduced (page 13).

**We have written out the PheWAS acronym in the Clinical results, rather than in the Methods where it is a specific package we are alluding to. We hope that this sufficiently clears up any ambiguity.**

3. At the beginning of the discussion, you state “as this analysis would be challenging or impossible in most U.S. states.” Can you clarify? Is this due to the current hostility toward medical care for TGD folks? If so, this should be noted in the discussion.

**We apologize for the lack of clarity in this statement. What we were referring to is the unique nature of the Utah Population Database and linkage across multiple medical records as well as administrative records. It is our understanding that this is not common in other states. However, to avoid potential misunderstandings, we have removed this phrase.**

4. On page 18, you state “The significant increase in dissociative disorder in the TGD cohort follows previous research linking transphobia and gender dysphoria to dissociative symptoms.” It may also be important to directly name this as trauma sxs/stressors (e.g. CPTSD).

**We have added an explicit comment to link dissociation to trauma, as well as an explicatory citation.**

5. Further down on page 18, you state that “Further research is necessary to determine the links between neurodivergence and trans identity and how gender-affirming care can best accommodate and empower patients with autism and ADHD, as well as other mental health conditions shown to be elevated in this community.” There have been multiple studies on the impact of GAC on MH of patients. It would be helpful to note some of these here (e.g. Chen et al, 2023; Dutton et al 2024; Tordoff, 2022).

**We have added the following sentence and citations: “Gender-affirming care has been shown by previous studies to broadly improve mental health in TGD individuals,**[**(Chen et al. 2023; Cheung et al. 2024; Tordoff et al. 2022)**](https://paperpile.com/c/fTi8zS/BlJC+M9D8+a995) **and so future studies may integrate the status and extent of gender-affirming care when evaluating mental health outcomes.”**

**Thank you for this point, as it clarifies our intention of highlighting specific contexts of TGD patients pursuing GAC, by asserting what is already known regarding GAC improving MH.**

6. On page 21 you note, “Ideally, future studies will improve as sex and gender documentation are improved, and there will be greater power to evaluate cohort-specific healthcare needs and the protective factors that may result from affirming and competent care.” I wonder if it would be helpful to separate this idea out as a recommendation (along with the timeline of dx to see how GAC may impact dx), and to briefly discuss the potential challenges to this in the current political/legislative climate.

**We have rephrased to include a specific recommendation on patient EHR. Please see response to Reviewer 1, Comment 5 for commentary on the current political/legislative climate. We have endeavored to be restrained in our commentary and focus on the aspects most pertinent to this study, but agree with the reviewers that the current political landscape has profound implications for trans health, both at level of the individual as ascertained by this study, and at the level of the clinics and communities endeavoring to provide support to TGD populations.**
