# Supplementary material for: Unveiling health disparities: Diagnostic prevalences in a transgender cohort versus matched controls
Source: PLoS One. 2025 Aug 6;20(8):e0329849. doi: 10.1371/journal.pone.0329849 (PMC12327606; doi:10.1371/journal.pone.0329849)
Supplement: Attachment — Submitted filename: Response_to_Reviewers_auresp_2.docx [file pone.0329849.s007.docx]

Dear Dr. Turban,

Thank you for reviewing our revised manuscript PONE-D-25-11456 “Unveiling Health Disparities: Diagnostic Prevalences in a Transgender Cohort versus Matched Controls”.

We thank the reviewers for their time and care reviewing the revised manuscript, and are glad that the revisions and point-by-point responses seemed to have overall addressed concerns and questions. In response to the remaining suggestion by Reviewer 2:

*“One minor suggestion is that the issue of the missing sex variable should be in the limitations. The authors explain that the missingness was due to the choice of relying on birth certificate sex from the database. This clearly was a fraught choice (especially if there were other forms of sex data that could have been gathered). The authors can very briefly mention that the missing sex data was a limitation and that future research should gather multiple sex-related data in administrative records that would be used for triangulating patients' sex. This would be an important "lesson learned" for future researchers who may embark on a similar endeavor.”*

We have added the following section to the first paragraph of our limitations section:

“Lastly, we report only the birth certificate sex. Despite availability, we elected not to use sex documented from other available vital records or EHR due to the assumed wide variability in practice, policy, and individual choice in completion of those records regarding a reflection of natal sex (assigned sex at birth). We observed mixed sex designations across multiple sources of data, and thus defaulted to a singular source of birth certificate sex for consistency. However, this variable was missing for individuals born out of state. Future studies may benefit from developing reliable methods of triangulation to determine sex assigned at birth for individuals without a birth certificate.”

Thank you again for this opportunity to share our research.
